# Information load dynamically modulates functional brain connectivity during narrative listening

**DOI:** 10.1038/s41598-023-34998-9

**Published:** 2023-05-19

**Authors:** Rossana Mastrandrea, Luca Cecchetti, Giada Lettieri, Giacomo Handjaras, Andrea Leo, Paolo Papale, Tommaso Gili, Nicola Martini, Daniele Della Latta, Dante Chiappino, Pietro Pietrini, Emiliano Ricciardi

**Affiliations:** 1 NETWORKS, IMT School for Advanced Studies, Lucca, Italy; 2 Social and Affective Neuroscience (SANe) Group, MoMiLab, IMT School for Advanced Studies, Lucca, Italy; 3 Crossmodal Perception and Plasticity Laboratory, Institute of Psychology, University of Louvain, Louvain-La-Neuve, Belgium; 4 MoMiLab, IMT School for Advanced Studies, Lucca, Italy; 5 Department of Translational Research and Advanced Technologies in Medicine and Surgery, University of Pisa, Pisa, Italy; 6 Department of Vision & Cognition, Netherlands Institute for Neuroscience (KNAW), 1105 BA Amsterdam, The Netherlands; 7 Fondazione Toscana G. Monasterio, Pisa, Italy

**Keywords:** Complex networks, Network models

## Abstract

Narratives are paradigmatic examples of natural language, where nouns represent a proxy of information. Functional magnetic resonance imaging (fMRI) studies revealed the recruitment of temporal cortices during noun processing and the existence of a *noun-specific* network at rest. Yet, it is unclear whether, in narratives, changes in noun density influence the brain functional connectivity, so that the coupling between regions correlates with information load. We acquired fMRI activity in healthy individuals listening to a narrative with noun density changing over time and measured whole-network and node-specific degree and betweenness centrality. Network measures were correlated with information magnitude with a time-varying approach. Noun density correlated positively with the across-regions average number of connections and negatively with the average betweenness centrality, suggesting the pruning of peripheral connections as information decreased. Locally, the degree of the bilateral anterior superior temporal sulcus (aSTS) was positively associated with nouns. Importantly, aSTS connectivity cannot be explained by changes in other parts of speech (e.g., verbs) or syllable density. Our results indicate that the brain recalibrates its global connectivity as a function of the information conveyed by nouns in natural language. Also, using naturalistic stimulation and network metrics, we corroborate the role of aSTS in noun processing.

## Introduction

Narratives are paradigmatic examples of the richness of natural language, able to evoke multifaceted and immersive experiences that cannot be merely ascribed to the analysis of syntactic, semantic and prosodic information. Indeed, a gamut of collateral processes related to mental imagery, embodiment and emotional resonance spontaneously arise as stories unfold^1,2^. These multiple processes concurrently involved in narrative listening translate into the recruitment of a vast extent of brain areas^3^, spreading well beyond the canonical language network^4–6^.

In natural language, nouns represent the building blocks of sentences and from an ontogenetic standpoint are acquired earlier as compared to other word classes^7^. When referring specifically to concrete entities, nouns are promptly processed^8^, robustly activate image-based codes^9^ and engage several brain regions^10–12^ with a precise temporal signature^13,14^. Specifically, the processing of nouns relies on bilateral engagement of angular gyrus, a core region of the semantic network^15,16^ involved in short-term verbal memory and sentence comprehension^17^. In addition, brain areas pertaining to high-order visual networks, including the left fusiform gyrus^18^, are recruited selectively when participants listen to nouns^19^, presumably due to the intrinsic perceptual nature of these linguistic entities^11^. Moreover, posterior associative cortical areas, including the cuneus, precuneus and posterior cingulate/retrosplenial cortex, which are typically engaged during mental imagery^20^ and autobiographical memory (see for a review^21^), also demonstrate preferential responses to concrete nouns^22,23^. This functional overlap likely stems from the ability of nouns to elicit vivid mental representations^24^ as well as from their being grounded into sensorimotor experience^25,26^.

Furthermore, temporal cortical areas are crucial regions selectively and consistently engaged during processing of nouns, as compared to other words classes^27,28^. Nonetheless, the nature of this specific recruitment is still largely debated, as the preferential response to nouns could be due more to semantic aspects, rather than to grammatical categories *per se*^29^.

Altogether, the above findings indicate that nouns are fundamental to convey information in language. In addition, a recent study showed that brain areas activated by nouns were also intrinsically more connected at rest with each other as compared to other cortical modules, indicating that processing of nouns may substantially shape brain connectivity^30^.

While the spatial characteristics of this functional fingerprint have been outlined, it remains unknown whether and to what extent the processing of nouns alters the coupling between specific brain areas during listening to natural language. Indeed, the study of brain connectivity dynamics may be crucial to capture the time-varying features of natural language, as it unveils how the synthesis of information occurs across time^31,32^.

In the present study, we aim to test the hypothesis that the load of information carried by nouns modulates functional brain connectivity over time. Thus, we designed a naturalistic paradigm to investigate the impact of nouns on whole-brain and node-specific network metrics (i.e., degree and betweenness centrality) as derived from functional magnetic resonance imaging (fMRI), while participants listened to a narrative having the amount of nouns varying over time.

## Materials and methods

### Participants

Nineteen healthy individuals participated in our study: twelve were recruited for the fMRI experiment (4 M; mean age ± standard deviation: 25 ± 2 years), aimed at measuring brain connectivity during listening to an original written narrative, whereas seven (2 M; mean age ± standard deviation: 29 ± 3 years) were enrolled in a behavioral task to characterize the time-varying presence of concrete nouns in the same narrative.

All individuals participated voluntarily and gave their written informed consent to take part in the study after risks and procedures had been explained and retained the right to withdraw from the study at any time. This study was approved by the Ethical Committee at the University of Pisa, Italy (protocol 1616/2003), and the study was conducted in accordance with the Declaration of Helsinki. All participants were clinically healthy and had no history of any relevant medical, neurological or psychiatric morbidity nor drugs or alcohol abuse. Specifically, none of the individuals had ever received a diagnosis of language-related developmental disorders (e.g., dyslexia, specific language impairment, delay of language onset). The Italian version of the Edinburgh Handedness Inventory^33^ assessed the individual manual preferences and only right-handed subjects (average score: 89.61; score range: 70.4–100.0) were included in the fMRI sample.

### Stimuli and experimental paradigm

**Figure 1:**
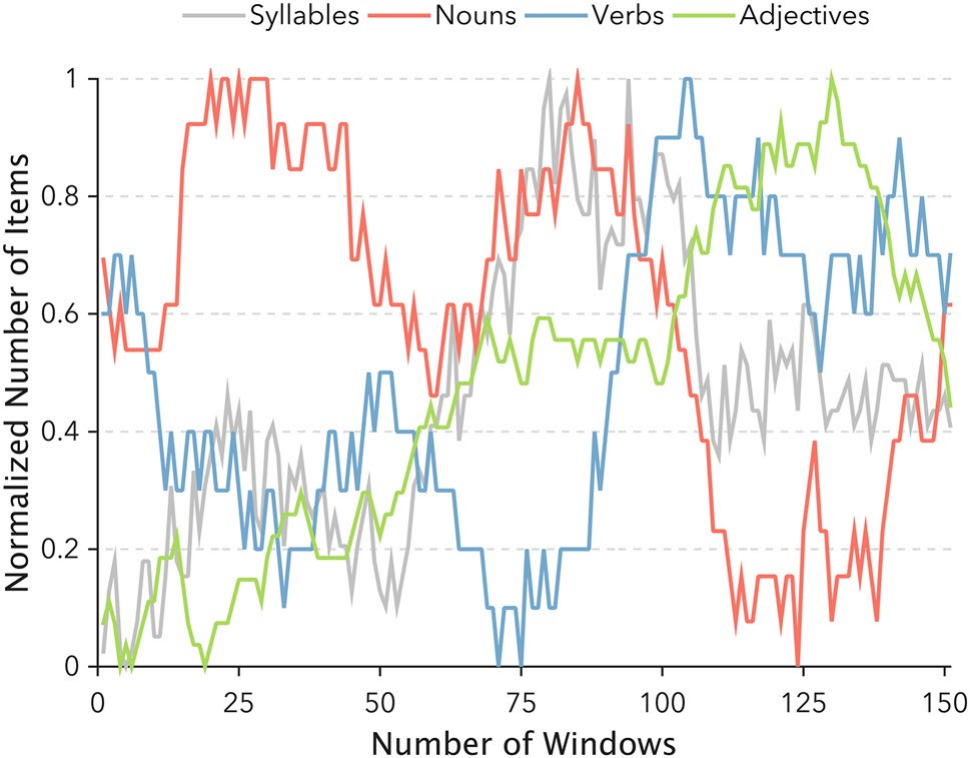
The figure depicts the distribution of syllables, nouns, verbs, and adjectives throughout our original narrative. A sliding-window procedure was applied to individual reports to obtain the overall number of concrete nouns included in 151 partially overlapping windows (60 s duration, 58 s overlap). To allow for a direct comparison of grammatical categories time-series, the number of items in each window is expressed in normalized units (i.e., the number of items for each window minus the minimum number of items across the whole time-series, all divided by the overall range).

Firstly, we conceived a six-minute story, having the number of nouns varying over time and characterized by a clearly distinct time-course for verbs and adjectives as well (Fig. 1). This short story was employed to estimate the relationship between information load (i.e., nouns density) and the interplay between distinct brain regions. Indeed, the specific temporal profile of nouns allowed us to correlate their presence with measures of brain network dynamics. In conceiving the narrative, we aimed at introducing smooth and slow transitions in the density of nouns that could be captured with a sliding window approach, rather than having brief periods of the story rich in nouns interleaved with segments with no nouns. The number of syllables also varied during the story (Fig. 1), albeit with a different profile, so as to disentangle changes in brain connectivity induced by nouns from those simply related to words length, a low-level marker of the entity of stimulation in a given time.

In addition, for our original written narrative, we particularly selected concrete nouns depicting common natural and urban landscapes, objects and human portraits (e.g., face, body parts) through perceptually rich scenarios, resembling everyday-life experiences, in order to favor mental imagery processes^34^. For the same reason, we decided to not include abstract nouns in the narrative, as they are less imageable than concrete nouns^35^.

The narrative (see Supplementary Information for English and Italian transcriptions) was recorded by one of the authors in a quiet room using a USB microphone (Behringer C-1U; 40–20,000 Hz, 130db max SPL) connected to a 13" MacBook Air ™ (Apple Inc.) running the Audacity v2.1.0 software (http://www.audacityteam.org). Sampling frequency of the recording was 48,000 Hz and a compressor filter (threshold = − 50 db; attack time = 0.2 s; release time = 1 s) was applied to achieve constant output volume across the story.

To ensure that the characteristics we introduced in the narrative, based on Italian grammar rules, had a behavioral counterpart in an independent sample of listeners, we first split the whole narrative into 180 non-overlapping 2 s-long segments. Afterwards, participants were instructed to count the number of concrete nouns in each part. This tagging approach has been already successfully adopted in the literature on functional neuroimaging and naturalistic stimulation to derive regressors of portrayed^36^ and felt^37,38^ emotions or object categories (^39^ in movies, for instance. A sliding-window procedure was applied to individual reports to obtain the overall number of concrete nouns included in 151 partially overlapping windows (58 s overlap), each lasting 60 s. The substantial overlap between neighboring windows determined smooth transitions between consecutive timepoints in the obtained timeseries (i.e., high correlation between adjacent windows), without precluding, however, the possibility to investigate temporal dynamics of the narrative. The agreement between reports was assessed using Spearman’s ρ and single-subject data were averaged together to derive a behavioral description of the varying amount of concrete nouns throughout the narrative. To ensure the validity of our stimulus, the significance of the correlation between behavioral reports and the varying amount of nouns in the narrative was also computed through a non-parametric permutation approach (see below for details).

The original continuous version of the story was employed in the fMRI part of the study, where participants were asked to attentively listen to the narrative and to picture the content of what it was described. Then, the very same sliding-window procedure was employed to study brain network reconfigurations during listening.

### MRI data acquisition and preprocessing

Brain activity elicited by listening to the original written narrative was recorded using Philips 3 T Ingenia scanner, equipped with a 8 channels phased-array coil, and a gradient recall echo-echo planar (GRE-EPI) sequence with the following acquisition parameters: TR/TE = 2000/30 ms, FA = 75°, FOV = 256 mm, acquisition matrix = 84 × 82, reconstruction matrix = 128 × 128, voxel size = 2 × 2 × 3 mm^3^, 38 interleaved axial slices (partial brain coverage, excluding the cerebellum), 190 total volumes, 6 min and 20 s overall scan time. A ten-second period of silence was added before the beginning of the narrative, and a silent period of the same duration was added after the story's ending. Three-dimensional high-resolution acquisition of the brain was also collected using a magnetization-prepared rapid gradient echo (MPRAGE) sequence (TR/TE = 7.07/3.21, FA = 9°, FOV = 224 mm, acquisition matrix = 224 × 224, voxel size = 1 × 1 × 1 mm^3^, 156 axial slices).

Data were analyzed using FSL v.5.0.9^40^ and MATLAB 2015a (v8.5.0) and for each subject preprocessing steps included: correction of slice time acquisition (ascending interleaved pattern) by Fourier-space time-series phase-shifting procedure and compensation of head movements by registering each brain volume to the computed average of the timeseries (MCFLIRT), with rigid body transformations (six degrees of freedom). Afterwards, the estimated six motion parameters were used to rule out residual confounds linked to subject movement during fMRI acquisition, which may lead to spurious results in connectivity studies^41^. Therefore, we computed an aggregated measure—framewise displacement (FD; fsl_motion_outliers)—that highlighted timepoints affected by excessive motion. We windowed this metric using a procedure identical to the one adopted for the behavioral data analysis (151 partially overlapping windows, each lasting 60 s with an overlap of 58 s) and the resulting single-subject timeseries were averaged to create a group-level timecourse of head motion. FD metric was also employed to generate a regressor for each motion spike (threshold ≥ 0.3; mean number of affected timepoints across subjects was 1.26%), then added as confound to the GLM at single-subject level (i.e., spike regression method^42^).

Spatial smoothing was applied employing a Gaussian kernel of FWHM 6 mm and grand-mean intensity scaling of the entire dataset (i.e., single multiplicative factor) was adopted to normalize the signal. In addition, high-pass temporal filtering ensured the correction of slow drifts in hemodynamic signal with a Gaussian-weighted least-squares straight line fitting (sigma = 50 s).

Brain extracted and preprocessed GRE-EPI sequences were fed into a GLM model, having as regressors of no-interest the six head motion parameters and motion spike regressors. The residuals of this linear regression model represented brain activity cleaned from motion- and scanner-related artifacts for each subject and served as input to compute brain connectivity measures.

The obtained timeseries were then linearly transformed to match the 2 mm Montreal Neurological Institute (MNI) 152 standard space using 12 degrees of freedom and trilinear interpolation. Craddock’s resting state functional parcellation^43^, originally comprising 100 regions of interest (ROIs), was used to extract brain hemodynamic activity during narrative listening. Regions that were not present in each subject due to brain coverage (ROIs Z coordinate range in standard space: − 36 to + 68; a full list of regions is provided in the shared code) limitations were not included in the analyses. Thus, we considered 81 ROIs from the original one hundred. Hemodynamic signal was extracted and averaged from 6 mm radius spheres located at the center of gravity of each ROI (network nodes), ultimately generating 81 timeseries of 180 timepoints each, having removed the 20 s of rest. Because we aimed at studying brain connectivity using a sliding window approach, which inherently introduces temporal smoothing, we decided not to convolve fMRI activity with the canonical hemodynamic response function.

Functional connectivity dynamics approach^44^ was used to capture brain network reconfigurations through time. A sliding-window procedure, identical to the one used for the behavioral part of the study, was applied to each of the 81 timeseries, resulting in 151 partially overlapping windows. The window duration and overlap (60 s duration; 58 s overlap) were chosen based on the paper by Hansen et al.^44^. For each participant, the 81 timeseries were pairwise correlated using Pearson’s coefficient within each window, so as to produce 151 correlation matrices. These single-subject correlation matrices were then averaged across individuals and thresholded using r = 0.2 obtaining 151 group-level time-varying network configurations, where nodes represent anatomical brain regions and edges the functional connections among them (i.e. the pairwise correlation between the BOLD time-series associated to each ROI). This threshold was selected in the following way: (i) we found for each correlation matrix (12 subjects for 151 windows = 1812 matrices) the maximum correlation value guaranteeing the absence of disconnected components in the associate functional network; (ii) we took the minimum value among the 1812 thresholds identified at point (i), w*; (iii) we thresholded the 1812 correlation matrices according to w*.

The resulting connectivity matrix is mathematically associated to an adjacency matrix, A, such that:

$$A \equiv \left( {a_{ij} } \right)_{1 \le i,j \le N} = \left\{ \begin{gathered} 1\quad {\text{if}}\;\rho_{ij} > w* \\ 0\quad {\text{otherwise}} \hfill \\ \end{gathered} \right.$$

where $\rho_{ij}$ represents the correlation value between the time-series associated to the brain areas i and j, N is the network size and w* the correlation threshold introduced before.

Two basic properties of network architecture related to centrality^45,46^ were then computed for each node: degree, a simple attribute reflecting the number of links and providing direct evidence of changes in the number of connections through time; betweenness centrality, the number of shortest paths connecting any other two nodes in the network and crossing the considered one, which highlights time-varying changes in network structure. Degree was computed for each window and node according to:

$$k_{i} = \sum\limits_{j = 1}^{N} {a_{ij} }$$

where $a_{ij}$ i is the *ij*-th element of the adjacency matrix *A* and *N* the total number of network nodes.

**Figure 2:**
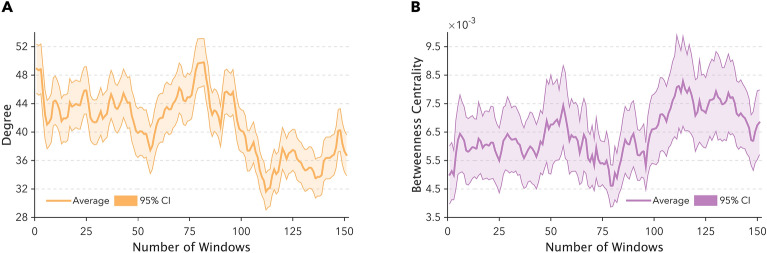
This figure represents the timecourse for the two network measures designated to track changes in whole-brain connectivity during narrative listening. Panel (**A**) shows the timeseries for degree, an attribute reflecting the number of neighbors node averaged between all the 81 ROIs, whereas panel (**B**) summarizes the temporal profile of betweenness centrality, a marker of whether a node participates in short paths, once again averaged across all the regions. Orange and purple solid lines represent the average timecourse and shaded area reports the 95% confidence interval.

Betweenness centrality was estimated according to:

$$b_{i} = \sum\limits_{s \ne i \ne t} {\frac{{\sigma_{st} \left( i \right)}}{{\sigma_{st} }}}$$

where $\sigma_{st}$ is the number of the shortest paths connecting the brain areas (i.e., the network nodes) s and t, while $\sigma_{st} \left( i \right)$ counts the number of shortest paths connecting the brain areas s and t and passing through the brain area i. It is worth to notice that the betweenness centrality depends on the number of pair of nodes. Therefore, the quantity can be normalised dividing by the number of pair of nodes not including node i, (N − 1)(N − 2)/2, such that the quantity belong to the range [0,1]. Aggregate measures of degree and betweenness centrality were obtained by averaging these two properties across the 81 ROIs (Fig. 2).

The association between network measures (i.e., degree and betweenness centrality) and the amount of concrete nouns across 151 windows was measured using Spearman correlation coefficient (ρ), and statistical significance was assessed using surrogate time-series. Briefly, 1000 surrogates were generated by randomizing the Fourier phases (i.e., iterative amplitude adjusted Fourier transform) of the noun time-series, and then correlated with the time-varying profile of the whole-network and the node-specific degree and betweenness centrality, so as to obtain a null distribution of ρ coefficients^47^. Importantly, the surrogates retained the same power spectrum, mean and standard deviation as the original time-series. To adjust the threshold of statistical significance for the number of comparisons, we used the family-wise error rate correction (FWEc) proposed by Nichols and Holmes^48^. The FWEc null distributions were obtained by computing the maximum (for the right FWEc tail) and the minimum (for the left FWEc tail) of surrogate correlations across ROIs. The FWEc *p* value of the original (unpermuted) relationship was estimated as its position in the right (if the unpermuted correlation was greater than zero) or the left (if the unpermuted correlation was smaller than zero) FWEc distribution tail. The analyses were repeated for other grammatical categories (i.e., verbs and adjectives) as well as for syllables.

## Results

### Behavioral experiment

**Figure 3:**
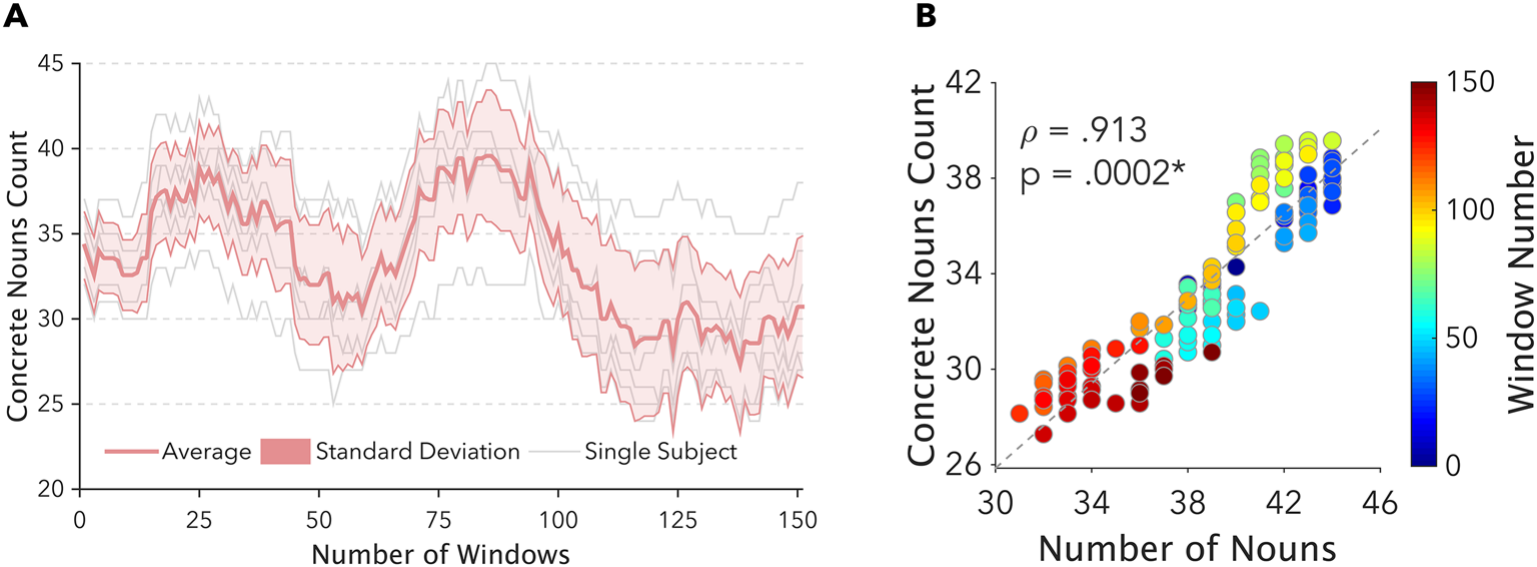
Panel (**A**) depicts the results for the behavioral experiment: here, the originally written narrative was split into 2 s-lasting segments and subjects were asked to count the number of concrete nouns in each part. The thick red line shows the timecourse of nouns density averaged across all subjects, while the red shaded area represents the standard deviation for this estimate and grey thin lines indicate single-subject data. The similarity between this behavioral estimate of nouns distribution over time and the number of nouns is shown in Panel (**B**), reporting the significant positive correlation between these measures (*p* = 0.0002). For each dot, color codes window number.

Seven participants were asked to count and report the number of concrete nouns in each of the narrative segments while listening to the story. The average agreement between single-subject data, considering windowed timeseries, (Fig. 3A) was ρ = 0.7861 (95% CI: 0.7107–0.8615) and the behavioral count of concrete items significantly correlated with the actual presence of nouns throughout the story (ρ = 0.913; *p* value = 0.0002; Fig. 3B). This ensured the validity of the nouns distribution that we defined when the narrative was conceived.

### Whole-brain connectivity results

Whole-brain connectivity results referred to degree and betweenness centrality computed and averaged across the 81 ROIs. We considered changes in these measures as time-varying reconfigurations of the whole brain network as function of information load, reflecting local (i.e., degree) and global (i.e., betweenness centrality) properties, respectively.

**Figure 4:**
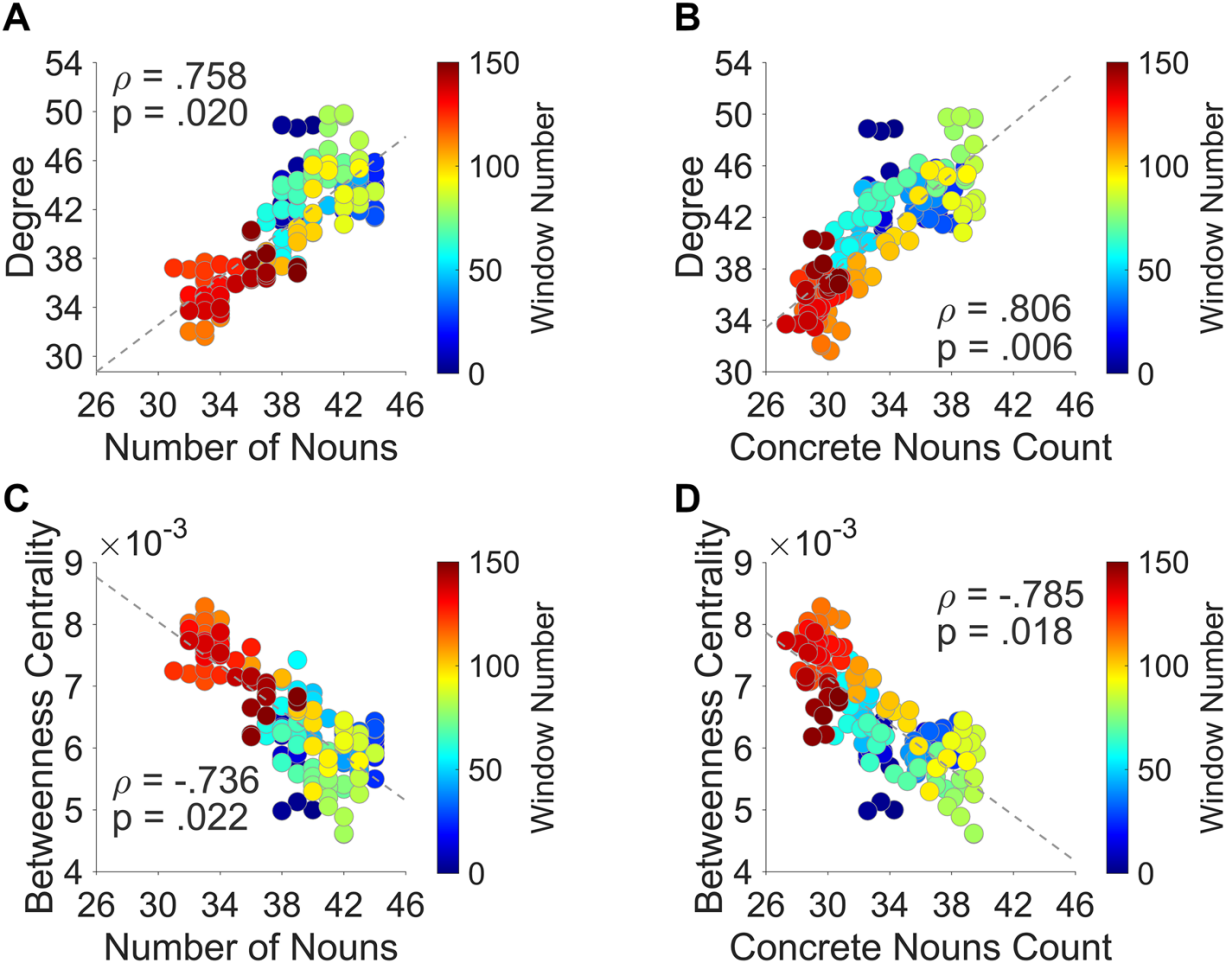
The figure reports the association between whole-brain connectivity measures and the profile of nouns density over time. Panel (**A**) represents the positive correlation between degree and the number of nouns included in our original narrative. Panel (**B**) depicts the relationship between the degree and the number of concrete nouns obtained through behavioral estimates. Panel (**C**) refers to the negative correlation between betweenness centrality and the number of nouns included in our original narrative whereas panel (**D**) depicts the association between this network measure and the number of concrete nouns obtained through behavioral estimates. All the reported *p* values reach statistical significance and survive correction for multiple comparisons according to the FWEc. For each dot, color codes window number.

Firstly, we found a significant positive correlation between the density of nouns in the narrative and the mean degree (ρ = 0.758; *p* value = 0.020; Fig. 4A). This translated into an increment of whole brain functional connectivity as the number of nouns to be processed increased. The same relationship was observed when looking at the behavioral count of concrete nouns throughout the narrative (ρ = 0.806; *p* value = 0.006; Fig. 4B).

Secondly, we found a significant negative correlation between mean betweenness centrality and the amount of nouns in the story (ρ = − 0.736; *p* value = 0.022; Fig. 4C), highlighting that as the number of nouns to be processed decreased, a reinforcement of central nodes of the network occurred, while functional connections to more peripheral regions are trimmed. The same association was also revealed when considering the behavioral count of concrete nouns performed by an independent sample of subjects (ρ = − 0.785; *p* value = 0.018; Fig. 4D).

Importantly, findings for the relationship between information load and whole-brain network measures cannot be merely ascribed to time-varying changes in syllables rate (mean degree: ρ = 0.043; *p* value = 0.956; mean betweenness centrality: ρ = − 0.038; *p* value = 0.950) or physiological confounds related to head motion (FD versus mean degree: ρ = 0.367; *p* value = 0.294; FD versus mean betweenness centrality: ρ = − 0.326; *p* value = 0.366).

In addition, verbs related positively to the average betweenness centrality (ρ = 0.669; *p* value = 0.016) and negatively to the average degree (ρ = − 0.676; *p* value = 0.020). These results are well explained by the negative association between the number of nouns and the presence of verbs in the narrative (ρ = − 0.683). As far as adjectives were concerned, neither the whole-brain average degree nor the average betweenness centrality correlated significantly with the number of adjectives (ρ = − 0.594; *p* value = 0.160 and ρ = 0.582; *p* value = 0.164, respectively).

### Node-specific connectivity results

**Figure 5:**
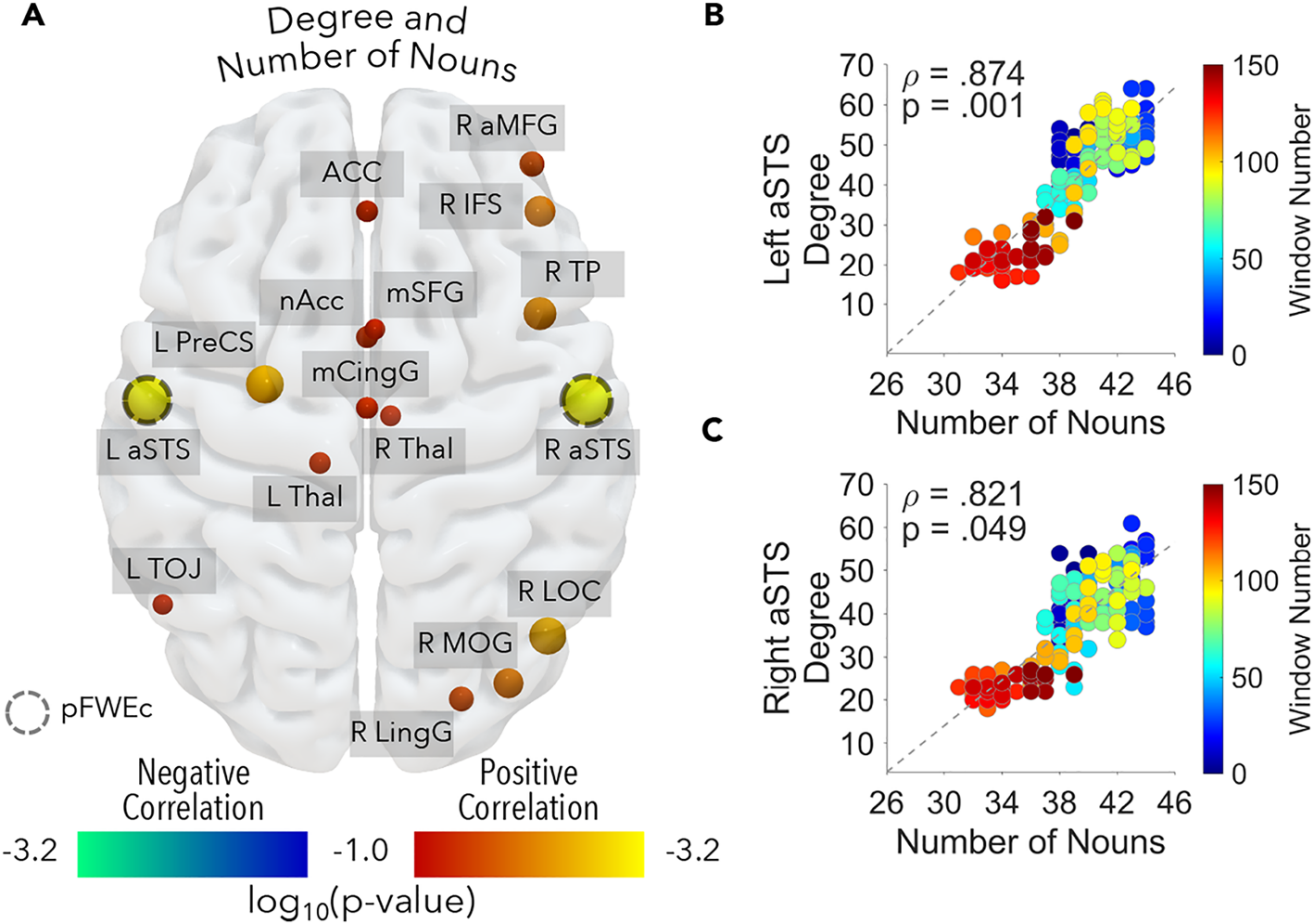
The figure reports the association between node-specific connectivity measures and the profile of nouns density over time. Panel (**A**) shows individual regions positively (i.e., log_10_ *p* value; red-yellow color map) or negatively (i.e., log_10_ *p* value; blue-green color map) associated to the timecourse of nouns (*p* < 0.05 uncorrected), according to the degree. The larger the sphere is, the higher the correlation; dashed outline indicates regions surviving the correction for multiple comparisons (*p* < 0.05 FWEc corrected): anterior portion of the left and right superior temporal sulcus. Panel (**B**) and (**C**) show the positive correlation between the degree of these two regions and nouns density over time. For each dot, color codes window number. R aMFG = right anterior portion of the middle frontal gyrus; ACC = anterior cingulate cortex; R IFS = right inferior frontal sulcus; R TP = right temporal pole; mSFG = medial portion of the superior frontal gyrus; nAcc = nucleus accumbens; L PreCS = left precentral sulcus; mCingG = middle part of the cingulate gyrus; R Thal = right thalamus; L aSTS = left anterior portion of the superior temporal sulcus; R aSTS = right anterior portion of the superior temporal sulcus; L Thal = left thalamus; L TOJ = left temporo-occipital junction; R LOC = right lateral occipital cortex; R MOG = right middle occipital gyrus; R LingG = right lingual gyrus; R SFS = right superior frontal sulcus; R aINS = right anterior insular cortex; L pMFG = left posterior portion of the middle frontal gyrus; L OFC = left orbitofrontal cortex; R PreCG = right precentral gyrus; L PostCG = left postcentral gyrus.

When considering network measures of each ROI, we found a significant positive correlation between the amount of nouns and the degree of the anterior portion of the superior temporal sulcus (Left aSTS: ρ = 0.874; FWEc *p* value = 0.001; Right aSTS: ρ = 0.821; FWEc *p* value = 0.049; Fig. 5A, B, C). Concerning the betweenness centrality, no association survived the correction for multiple comparisons (all FWEc *p* values > 0.05). These results were confirmed using behavioral reports of noun density collected in independent participants: the degree of both left (ρ = 0.856; FWEc *p* value = 0.006) and right (ρ = 0.790; FWEc *p* value = 0.015) aSTS related positively to the number of nouns.

Interestingly, when testing the association between all other grammatical categories (i.e., verbs and adjectives) and ROI-specific network metrics, we found no correlations surviving the FWE-corrected statistical threshold (all FWEc *p* values > 0.05).

Lastly, also the number of syllables did not correlate with any network measures of any brain region (all FWEc *p* values > 0.05).

## Discussion

Given the high relevance of nouns in conveying information, several investigations have focused on the localization of *noun-specific* brain areas. While there is a notable consensus regarding the involvement of temporal cortical areas in noun processing, a characterization of network properties of these regions during language comprehension in naturalistic conditions is still lacking. To examine the impact of information load carried by nouns on brain connectivity dynamics, here we measured hemodynamic activity of healthy individuals while they attentively listened to an original written narrative, having nouns density varying through time. Network measures explored the relationship between whole-brain and node-level connectivity and the number of nouns as a proxy of the amount of information to be processed moment-by-moment.

Our results demonstrate that nouns timecourse was positively associated to the average number of functional connections (i.e., pairwise correlations between BOLD time series of ROIs) across regions, indicating an increment in connectivity as information load increased. In addition, nouns were negatively related to network betweenness centrality, suggesting that peripheral connections were trimmed as information decreased. Further, when considering network properties of individual nodes, the number of functional links of the anterior superior temporal sulcus (aSTS) was positively associated with nouns. Importantly, changes in the connectivity of aSTS cannot be merely explained by the timecourse of other parts of speech, such as verbs or adjectives, and do not depend on a generic word density effect, as we found no significant correlation between aSTS network metrics and the number of syllables. Taken together, these findings support our original interpretation that nouns are a proxy of information load and reveal how nouns impact whole-brain and node-specific properties of brain connectivity during naturalistic stimulation, emphasizing the role of the anterior temporal cortex.

### Effects of information load on whole-brain and node-specific connectivity

The presence of nouns significantly altered the amount and characteristics of whole-brain connectivity, with the increase in the amount of information to be processed fostering the growth in the number of links, connecting especially peripheral nodes.

Specifically, the degree is a graph-theoretical measure of brain regions connectivity, which assesses sharing of information among distinct areas and an increment in this measure is interpreted as a boost in workload^49^. Information integration is eased by nodes with a higher number of functional connections, thus networks able to rapidly and efficiently process stimuli are generally characterized by a large number of highly connected nodes^50,51^. Successful performances at various highly-engaging cognitive tasks do rely on the formation of a widespread but integrated network^52^, while simpler tasks do not require the same level of integration^52,53^. In line with this, the brain maintains a segregated state during rest conditions, and becomes more integrated as the cognitive demand of tasks increases, with highest levels particularly related to working-memory and language domains^54^.

Overall, these studies point out that dynamic changes in brain functional connectivity are associated with distinct behavioral performances and track variations occurring in the surrounding environment^55^. In our study, we manipulated the amount of information load over time and found a positive correlation between nouns density and the growth of a widespread but integrated network. Hence, bearing in mind previous evidence of a positive relationship between cognitive demand and network density, we conclude that nouns represent a signature of information load in natural language processing. This is particularly true since measures of words length, a low-level marker of the entity of stimulation in a given time, did not correlate with variations in the dynamic interplay among brain regions.

The anterior portion of the superior temporal sulcus stood out when considering the number of connected nodes: indeed, this region became more connected as information load increased. Interestingly, in addition to playing a crucial role in the processing of nouns^56^, aSTS is involved in other processes as well, such as social perception^57^ or emotion^58^. Social and emotional aspects are often crucial in narratives, as books and novels primarily describe people’s (mis)fortunes. Regarding the narrative used in this study, we describe a social situation in the very last part of the story (please refer to the Supplementary Information). Also, during this part, the density of nouns reaches its minimum. The existence of a positive relationship between noun density and aSTS connectivity suggests that—in the present case—information load, rather than emotional or social aspects, has shaped network properties of these regions^59^.

### Nouns processing from a network perspective

Nouns represent a fundamental linguistic entity and their processing has been associated to the recruitment of a specific pattern of brain regions. Seminal studies, indeed, recognized a reliable involvement of temporal lobe structures in nouns processing and damages affecting these cortical areas have been reliably implicated in a selective impairment for nouns^60,61^. For instance, the processing of nouns is extensively impaired in semantic dementia, including the well-known reversed concreteness effect^62^, due to the underlying atrophy pattern involving temporal structures^61^. Furthermore, patients affected by fluent aphasia, generally suffering lesions in the temporal lobe territories, show relevant naming impairments for nouns rather than verbs^63^. Nonetheless, deficits in nouns processing following brain damage are less frequent, as compared to impairments in verbs^60,63^, thus indicating the potential existence of a distinct brain network dedicated to nouns.

Nonetheless, it should be noted that other parts of speech, such as verbs and adjectives, often contribute to the full understanding of the discourse, adding relevant context to nouns.

The vast majority of studies adopted a localizationist approach or relied upon the observation of pathological conditions to draw inferences on the specific signature of nouns processing in the brain. By way of illustration, canonical fMRI studies are used to dissect the phenomenon of interest in its fundamental components, reduce its complexity, and control possible intervening effects. This approach is adopted in different contexts, including investigations on language (see for instance^4^). Such a procedure is effective in understanding isolated processes, but has some limitations in drawing conclusions regarding global aspects of perception and cognition, limiting de facto our understanding of brain functioning in real-life situations. Conversely, the use of naturalistic and dynamic stimuli, such as movies^38,64^, continuous speech or stories^65^ in the fMRI experimental setting can provide a characterization of the dynamic interplay among brain regions that likely occur in ecological situations. Furthermore, only recent advances in the computation of network dynamics made it possible to investigate noun processing under naturalistic conditions, introducing a holistic perspective that can pave the way to new and valuable insights^66^.

In line with this, our study demonstrates that naturalistic stimulations mimicking real-language experiences are necessary to properly capture the dynamic nature of brain regional interactions during language comprehension.

Nonetheless, our fMRI paradigm is not immune to criticism. For instance, since in the original written narrative we used mainly concrete nouns referring to landscapes, objects and human features, we did not unmistakably characterize them as a grammatical class, and for this reason their effects on brain connectivity could be driven more by semantic features (for a review on the topic see^29^). Indeed, as some evidence pointed out, there seems to be no difference in the processing of nouns and verbs when they are semantically matched^67^. However, it is important to emphasize that this approach, aimed at artificially matching semantic characteristics of nouns and verbs, does not consider the importance of universal prototypes in language, where the subject of discourse is generally a noun referring to a person or a thing, whereas verbs usually refer to activities^27^.

In conclusion, the results of the present study expand the current knowledge on the brain correlates of noun processing, by providing the first demonstration of how, following an increase in the amount of information conveyed by nouns in natural language, the brain recalibrates its global connectivity properties, forming novel functional connections also with more peripheral regions. Furthermore, by highlighting network properties of the anterior superior temporal cortex, our findings corroborate the central role of this region in noun processing.

## Supplementary Information

Supplementary Information.

## Data Availability

Raw data used and/or analysed in the current study cannot be shared due to privacy reasons. Nevertheless, we provide upon request (rossana.mastrandrea@imtlucca.it) the MATLAB code and the pre-processed average network metrics and behavioral data sufficient to reproduce the present findings as supplementary materials.
