# Supplementary material for: Information load dynamically modulates functional brain connectivity during narrative listening
Source: Sci Rep. 2023 May 19;13:8110. doi: 10.1038/s41598-023-34998-9 (PMC10199005; doi:10.1038/s41598-023-34998-9)
Supplement: Supplementary file 1 — Supplementary Information. [file 41598_2023_34998_MOESM1_ESM.pdf]

Supplementary Information

Information load dynamically modulates functional brain connectivity during narrative listening

Rossana Mastrandrea[a+*], Luca Cecchetti[b*], Giada Lettieri[b,c], Giacomo Handjaras[d], Andrea Leo[e], Paolo Papale[d,f], Tommaso Gili[a], Nicola Martini[g], Daniele Della Latta[g], Dante Chiappino[g], Pietro Pietrini[d], Emiliano Ricciardi[d]

[a] NETWORKS, IMT School for Advanced Studies Lucca, Lucca, Italy
[b] Social and Affective Neuroscience (SANe) Group, MoMiLab, IMT School for Advanced Studies Lucca, Lucca, Italy.
[c] Crossmodal Perception and Plasticity Laboratory, Institute of Psychology, University of Louvain, Louvain-la-Neuve, Belgium
[d] MoMiLab, IMT School for Advanced Studies Lucca, Lucca, Italy
[e] Department of Translational Research and Advanced Technologies in Medicine and Surgery, University of Pisa, Pisa, Italy
[f] Department of Vision & Cognition, Netherlands Institute for Neuroscience (KNAW), 1105 BA Amsterdam, Netherlands.
[g] Fondazione Toscana G. Monasterio, Pisa, Italy

[+] Corresponding author; * these two authors contributed equally

English transcription of the narrative

The high mountains surrounded the waters of the big lake. On a beautiful spring day, the sun made the color of the peaks bright, while the water moved calmly. Further down, the green fields of the hills were dug by small streams that ran to the valley to throw themselves into the lake. At the center, in the middle of the water, you could see a small uninhabited island, surrounded by a dark beach and large gray boulders. At the foot of the hills there were some houses with red roofs and, a little further on, right on the shore of the lake, stood one of the largest villages. Small sailboats continuously sailed from the harbour, that moving along the side of the lake, skirted the main square, where the church and the town hall stood, with the big clock and flag. The houses, with pointed roofs, were arranged neatly along the streets that led to the shores of the lake. Not far away, further from the shore, stood another paved square with light and dark stones and with a large circular fountain in the center and some empty benches. Farther away from the village, near the top of the hill a stone house had been built with a large garden full of fruit trees and flowers. There were firs and beeches, and besides the peach and apple trees, there were a series of yellow roses, alternating with colorful violets and large daisies with white petals. The garden was closed by a stone wall that ended with a large wrought iron gate. From here, a gravel road led to the front door of the house.
After the dark wooden entrance door, a long, narrow, sparsely furnished corridor with many doors opened. On the right side was the study with a very large desk and a bookcase that ran along two sides. On the same side, in the next doors there were two brightly colored bedrooms. The first was all pink with a four-poster bed and a series of shelves with dolls with long and gaudy clothes. The second had green walls and the brown bed had a cover that resembled a tent. At the end of the corridor, there was a large heavenly bathroom with two marble sinks and a corner bath. On the left side, instead, a very large room opened with two sofas placed in front of a big fireplace and

an oval table, illuminated by the crystal chandelier. The living room communicated with a small kitchen, characterized by a dark marble top, light wooden furniture and a rectangular table surrounded by four white chairs. Still facing the corridor, in the door next to the hall, there was the master bedroom with a large black wrought-iron bed and a large dark wooden wardrobe. In front of the thick fabric curtain of the window was an armchair, a dark-framed mirror, and a small table with a small glass lamp above it. From the bedroom it was possible to access a small pale pink bathroom that had a large shower and a wardrobe that contained colored towels and bottles.

On that day, a group of people gathered around the large table in the hall. There was a thirty-year-old woman with a thin body, pale skin and long hair gathered in an elaborate hairstyle, which let a tuft fall over her eyes. She had a smiling face and his green eyes gleamed at the sides of his thin and regular nose. Her right hand was resting on the shoulder of a man seated not far from her. He was tall and rather corpulent, a few years older, his hair was curly and black, framing a round, beardless face and eyes of the same deep color. Two old ladies spoke aloud, the first one with a red face, long white hair in a ponytail, and dark eyes, hidden by little golden-rimmed glasses. The second one was very thin, brunette with short hair and a big aquiline nose. Next to the women, listening to the conversation, sat a seven-year-old girl with a round face and rosy cheeks. Her hair was dark brown, gathered in two braids that fell on her shoulders and left her ears uncovered, adorned with two small gold earrings. Next to the child was a short, plump old man who wore a pair of thick brown glasses on his big nose. Beside him, a tall, distinguished old man was curling his thick white mustache, which mostly covered his thin lips. In the middle, there was a very thin, tall child with dark, curly hair. The always smiling mouth hid two straight rows of very white teeth.

Everyone seemed happy and wore small caps in colored paper: it was the birthday party of the child who, standing on the chair, held a light blue balloon in one hand and in the other one a gift bag lined with bright paper. In front of him there was a cake with four blue candles on it in a row. The paper cap fell on one side of his head and he held a colored trumpet in his mouth.

Italian transcription of the narrative

Le alte montagne circondavano le acque del grande lago. In una bella giornata primaverile, il sole rendeva il colore delle cime brillante, mentre l’acqua si muoveva calma. Più in basso, i prati verdi delle colline erano scavati da piccoli ruscelli che correvano fino alla valle per gettarsi nel lago. Al centro, in mezzo all’acqua si scorgeva una piccola isoletta disabitata, contornata da una spiaggia scura e da grossi massi grigi. Ai piedi delle colline si trovavano alcune case dai tetti rossi e poco più in là, proprio sulla riva del lago, sorgeva uno dei paesi più grandi. Dal porto salpavano continuamente delle piccole barche a vela, che muovendo lungo la sponda del lago, costeggiavano la piazza principale, in cui si trovavano la chiesa e il palazzo del comune, con il grande orologio e la bandiera. Le case, dai tetti a punta, erano disposte in modo ordinato lungo le vie che portavano alle sponde del lago. Poco distante, più lontano dalla riva, sorgeva un’altra piazza lastricata con pietre chiare e scure e con al centro una grande fontana circolare e alcune panchine vuote. Più lontano dal paese,

vicino alla sommità della collina era stata costruita una casa in pietra con un grande giardino ricco di alberi da frutto e fiori. C'erano abeti e faggi e oltre agli alberi di pesco e a quelli di mele, si trovavano una serie di rose gialle, alternate a viole coloratissime e grandi margherite con i petali bianchi. Il giardino era chiuso da un muretto in pietra che terminava con un grande cancello in ferro battuto. Da qui, una strada in ghiaia bianca, conduceva alla porta d'ingresso della casa.

Dopo la porta d'ingresso in legno scuro, si apriva un lungo e stretto corridoio scarsamente arredato e con molte porte. Sul lato di destra si affacciava lo studio con una scrivania molto grande e una libreria che correva lungo due parerti. Sullo stesso lato, nelle porte successive erano collocate due camerette dalle tinte vivaci. La prima era tutta rosa con un letto a baldacchino e una serie di mensole con bambole dai vestiti lunghi e sgargianti. La seconda aveva le pareti di colore verde e il letto marrone aveva una copertura che ricordava una tenda. In fondo al corridoio, si trovava un grande bagno celeste con due lavabi in marmo e una vasca angolare. Sul lato sinistro, invece si apriva una sala molto grande con due divani posti davanti a un grande camino e un tavolo ovale, illuminato dal lampadario in cristallo. Il salotto comunicava con una cucina piccola, caratterizzata da un piano in marmo scuro, i mobili in legno chiaro e un tavolo rettangolare circondato da quattro sedie bianche. Sempre affacciata sul corridoio, nella porta successiva alla sala, c'era la camera padronale con il grande letto in ferro battuto nero e un armadio ampio di legno scuro. Davanti alla tenda di tessuto spesso della finestra c'erano una poltrona, uno specchio con la cornice scura e un tavolino con sopra una piccola lampada di vetro. Dalla camera era possibile accedere a un piccolo bagno di colore rosa pallido che aveva una grande doccia e un armadio che conteneva asciugamani e bottigliette colorate.

Quel giorno, intorno al grande tavolo della sala era presente un gruppo di persone. C'era una donna di trent'anni dal corpo asciutto, pelle chiara e capelli lunghi raccolti in un'elaborata acconciatura, che lasciava cadere un ciuffo sugli occhi. Aveva il volto sorridente e i suoi occhi verdi brillavano ai lati del naso sottile e regolare. La sua mano destra era appoggiata sulla spalla di un uomo seduto poco distante da lei. Era alto e piuttosto corpulento, di qualche anno più vecchio, aveva i capelli ricci e neri che incorniciavano un viso rotondo e senza barba e gli occhi dello stesso colore profondo. Due signore anziane parlavano a voce alta, la prima aveva il volto arrossato, lunghi capelli bianchi, che portava raccolti, e occhi scuri, nascosti da piccoli occhiali dalla montatura dorata. La seconda era molto magra, castana con i capelli corti e un grosso naso aquilino. Accanto alle donne, intenta ad ascoltare la conversazione, sedeva una bambina di sette anni dal visetto rotondo e le guance rosee. Aveva i capelli lisci color castano scuro, raccolti in due trecce che cadevano sulle spalle e lasciavano scoperte le orecchie, adornate da due piccoli orecchini in oro. Vicino alla bambina era seduto un uomo anziano basso e grassottello che portava sul grande naso un paio di occhiali marroni dalle lenti molto spesse. Al suo fianco un uomo anziano, alto e distinto si stava arricciando i folti baffi bianchi che coprivano per gran parte le labbra sottili. In mezzo, c'era un bambino molto magro e alto, con i capelli scuri e ricci. La bocca sempre sorridente nascondeva due file dritte di dentini bianchissimi.

Tutti sembravano allegri e indossavano piccoli cappellini in carta colorata: era la festa di compleanno del bambino che, in piedi sulla sedia, teneva con una mano un

palloncino azzurro e con l’altra un pacco dono foderato con carta sgargiante. Davanti a lui c’era una torta con sopra quattro candeline blu disposte in fila. Il cappellino di carta gli cadeva su un lato della testa e in bocca reggeva una trombetta colorata.
